# An Exploration of the Roles of Ferric Iron Chelation-Strategy Components in the Leaves and Roots of Maize Plants

**DOI:** 10.3390/plants8050133

**Published:** 2019-05-18

**Authors:** Georgios Saridis, Styliani N. Chorianopoulou, Yannis E. Ventouris, Petros P. Sigalas, Dimitris L. Bouranis

**Affiliations:** 1 Botanical Institute, Cologne Biocenter, University of Cologne, D–50674 Cologne, Germany; g.saridis@uni-koeln.de; 2 Plant Physiology and Morphology Laboratory, Crop Science Department, Agricultural University of Athens, 75 Iera Odos, Athens 11855, Greece; yannisventouris@gmail.com (Y.E.V.); bouranis@aua.gr (D.L.B.); 3 Rothamsted Research, West Common, Harpenden, Hertfordshire AL5 2JQ, UK; petros.sigalas@rothamsted.ac.uk

**Keywords:** arbuscular mycorrhizal symbiosis, iron deficiency, iron homeostasis, sulfur deficiency, sulfur interactions, *Zea mays*

## Abstract

Plants have developed sophisticated mechanisms for acquiring iron from the soil. In the graminaceous species, a chelation strategy is in charge, in order to take up ferric iron from the rhizosphere. The ferric iron chelation-strategy components may also be present in the aerial plant parts. The aim of this work was to search for possible roles of those components in maize leaves. To this end, the expression patterns of ferric iron chelation-strategy components were monitored in the leaves and roots of mycorrhizal and non-mycorrhizal sulfur-deprived maize plants, both before and after sulfate supply. The two levels of sulfur supply were chosen due to the strong impact of this nutrient on iron homeostasis, whilst mycorrhizal symbiosis was chosen as a treatment that forces the plant to optimize its photosynthetic efficiency, in order to feed the fungus. The results, in combination with the findings of our previous works, suggest a role for the aforementioned components in ferric chelation and/or unloading from the xylem vessels to the aerial plant parts. It is proposed that the gene expression of the DMA exporter *ZmTOM1* can be used as an early indicator for the establishment of a mycorrhizal symbiotic relationship in maize.

## 1. Introduction

Iron is an essential element for plant growth and productivity. It participates in electron transfer reactions and is central to the function of heme- and Fe–S cluster-requiring enzymes; thus, iron is required for various cellular processes, including respiration, photosynthesis, sulfur assimilation, and nitrogen fixation [1]. Although soil contains abundant iron, its bioavailability is extremely low. Under aerobic conditions and in the physiological pH range, iron is mainly present as oxidized ferric (hydr)oxides, which are sparingly soluble and not available to plants [2]. Therefore, plants have developed sophisticated and tightly regulated mechanisms for acquiring iron from soil. Non-graminaceous plants reduce ferric iron to the more soluble ferrous form at the root surface, by exporting a number of metabolites (including organic acids, phenolics, flavonoids and flavins), inducing the expression of ferric-chelate reductase, and they transport the resulting ferrous ions across the root plasma membrane. In contrast, graminaceous plants use a chelation strategy to take up iron from their rhizosphere. They secrete phytosiderophore compounds from their roots, phytosiderophores solubilize iron in the soil, and then the roots take up the resulting ferric iron–phytosiderophore complexes [1]. Maize in particular biosynthesizes only the 2′-deoxymugineic acid (DMA) via DMA synthase (ZmDMAS1). DMA is secreted into the rhizosphere via a specific exporter (ZmTOM1), and then the ferric iron-DMA complex is taken up by the ZmYS1 transporter [3].

Once iron has entered the root symplast, it is transported through the root cortex in the form of ferrous iron–nicotianamine complexes. After entering the root pericycle, it can be loaded into the xylem for transport to the shoot’s sink tissues. The dominant form of iron in the xylem sap of non-graminaceous plants is ferric iron–citrate, whereas in graminaceous plants the bound iron forms may be ferric iron–citrate, along with ferric iron–phytosiderophores [4]. An important sink tissue for iron is the leaves, where it re-enters the symplast, is reduced to the ferrous form, and is again found as ferrous iron–nicotianamine.

During this “route of iron” within a graminaceous plant, it seems that ferric iron chelation-strategy components may also be present in the aerial plant parts. The expression of the DMA synthase gene in the leaves of rice and barley suggests a possible role(s) of DMA in leaf iron homeostasis, under various growth conditions and developmental stages. In rice shoot tissue, no expression of *OsDMAS1* was detected in the leaves of iron-sufficient plants, whereas under iron-deficient conditions, the *OsDMAS1* gene was specifically expressed in vascular bundles of the leaves [3]. 

Therefore, a plausible question arises as regards the role (or roles), if any, of those components in maize leaves. In a previous work of our group it was demonstrated that arbuscular mycorrhizal (AM) symbiosis impeded the expected iron deprivation responses in sulfur-deprived maize plants [5]. Sulfur deficiency has a negative impact on the iron homeostasis of all plants, due to the fact that the primary precursor of nicotianamine (i.e., the primary iron-chelating compound into the symplasm) is the sulfur-containing amino acid methionine. Nicotianamine is synthesized via the enzyme nicotianamine synthase (NAS), which uses S-adenosyl-methionine as a substrate molecule. For the graminaceous plants in particular, sulfur deficiency also exerts an impact on the iron uptake, since nicotianamine is the precursor compound of phytosiderophores. In this study, the gene expression patterns of ferric iron chelation-strategy components were monitored in the leaves (*ZmDMAS1*, *ZmTOM1* and *ZmYS1*) and roots (*ZmDMAS1* and *ZmTOM1*) of mycorrhizal and non-mycorrhizal sulfur-deprived maize plants, both before and after (24 and 48 h) sulfate supply.

## 2. Results

### 2.1. Expression Levels of ZmDMAS1 in the Leaves

**Figure 1:**
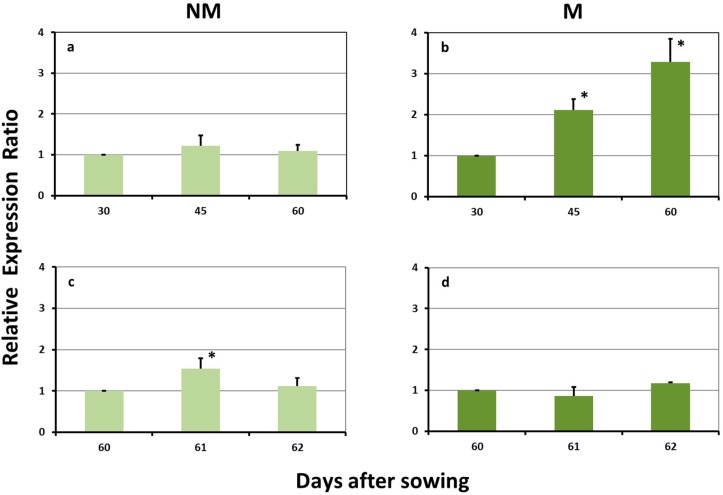
Expression of *ZmDMAS1* in the leaves of non-mycorrhizal (NM; **a**,**c**) and mycorrhizal (M; **b**,**d**) maize plants, (**a**,**b**) before and (**c**,**d**) after the sulfur supply, relative to the expression of ubiquitin. Bars show the mean of the biological replicates ± SE, asterisk (*) indicates the statistically significant difference between the sampling and the respective control at *p* < 0.05.

No significant difference in the expression levels of *ZmDMAS1* in the leaves of non-mycorrhizal (NM) plants was monitored during the long sulfur-deficient period of time. The sulfur supply on day 60 resulted in a transient upregulation of this gene 24 h after this supply, whilst afterwards the expression levels reverted to the previous values (Figure 1a,c). The leaves of mycorrhizal (M) plants showed a differential response, both during the sulfur-deprived as well as after the sulfur-repletion period. The expression levels were constantly increased during the first 60 days of the study. The addition of sulfate had no significant effect on the expression values, which remained unaffected on days 61 and 62 (Figure 1b,d). 

### 2.2. Expression Levels of ZmTOM1 in the Leaves

**Figure 2:**
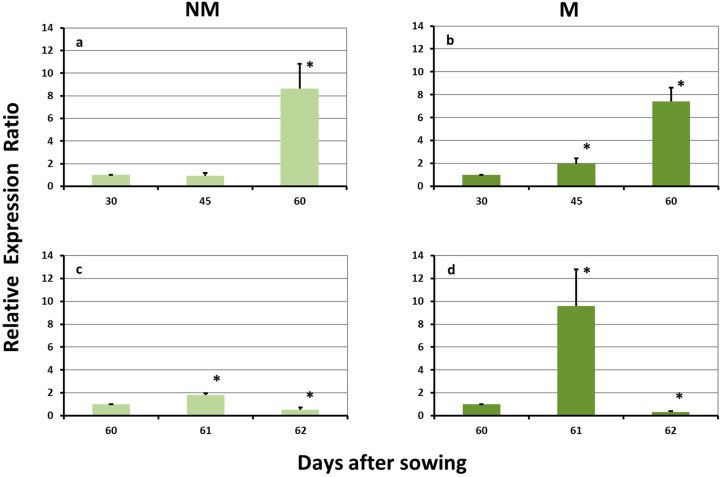
Expression of *ZmTOM1* in the leaves of non-mycorrhizal (NM; **a**,**c**) and mycorrhizal (M; **b**,**d**) maize plants, (**a**,**b**) before and (**c**,**d**) after the sulfur supply, relative to the expression of ubiquitin. Bars show the mean of the biological replicates ± SE, asterisk (*) indicates the statistically significant difference between the sampling and the respective control at *p* < 0.05.

A prolonged sulfur deprivation resulted in a strong overexpression of *ZmTOM1* in the leaves of NM plants on day 60. The influx of sulfate caused a further overexpression 24 h after the addition of sulfur. The second day after the sulfur supply, the expression levels decreased significantly (Figure 2a,c). *ZmTOM1* was also strongly upregulated in the leaves of M plants on day 60, but in this case, it was already upregulated from day 45. The sulfur supply induced a strong overexpression on day 61, followed by an equally strong downregulation on day 62 (Figure 2b,d). 

### 2.3. Expression Levels of ZmYS1 in the Leaves

**Figure 3:**
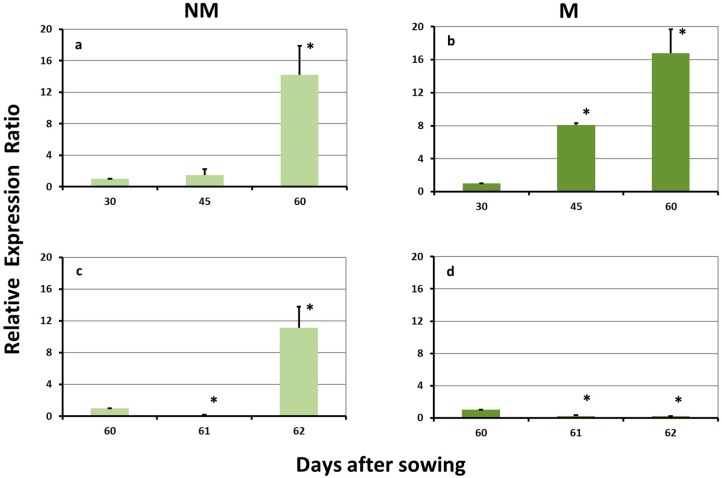
Expression of *ZmYS1* in the leaves of non-mycorrhizal (NM; **a**,**c**) and mycorrhizal (M; **b**,**d**) maize plants, (**a**,**b**) before and (**c**,**d**) after the sulfur supply, relative to the expression of ubiquitin. Bars show the mean of the biological replicates ± SE, asterisk (*) indicates the statistically significant difference between the sampling and the respective control at *p* < 0.05.

*ZmYS1* was strongly upregulated in the leaves of NM plants on day 60, before sulfur supply. The sulfur supply resulted in a transient downregulation on day 61, followed by a strong overexpression 2 days after the influx of sulfate (Figure 3a,c). The expression levels of this gene in the leaves of M plants increased constantly during the sulfur-deprived period and presented a strong overexpression on both sampling days. The addition of sulfate resulted in a strong downregulation on day 61, whilst the expression levels also remained reduced on day 62 (Figure 3b,d). 

### 2.4. Expression Levels of ZmDMAS1 in the Roots

**Figure 4:**
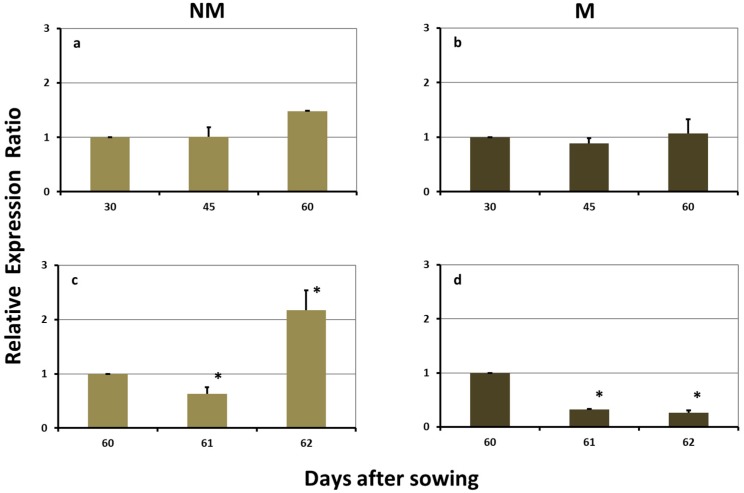
Expression of *ZmDMAS1* in the roots of non-mycorrhizal (**NM**; **a**,**c**) and mycorrhizal (**M**; **b**,**d**) maize plants, (**a**,**b**) before and (**c**,**d**) after the sulfur supply, relative to the expression of ubiquitin. Bars show the mean of the biological replicates ± SE, asterisk (*) indicates the statistically significant difference between the sampling and the respective control at *p* < 0.05.

No significant difference in the expression levels of *ZmDMAS1* was monitored in the roots of NM plants during the sulfur deprivation treatment. The sulfur supply induced a strong downregulation of the expression, followed by an overexpression 2 days after the sulfate influx (Figure 4a,c). No significant difference in the expression levels of *ZmDMAS1* during sulfur deprivation was also observed in the roots of M plants. The addition of sulfate caused a strong downregulation of this gene expression on day 61. The same reduced levels were also observed on day 62 (Figure 4b,d). 

### 2.5. Expression Levels of ZmTOM1 in the Roots

**Figure 5:**
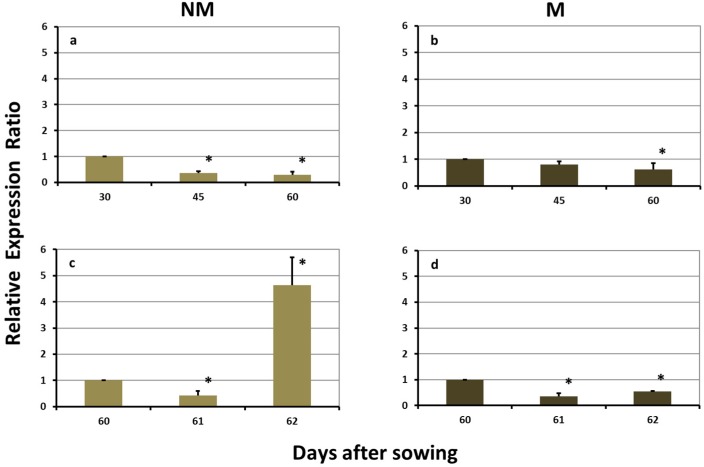
Expression of *ZmTOM1* in the roots of non-mycorrhizal (NM; **a**,**c**) and mycorrhizal (M; **b**,**d**) maize plants, (**a**,**b**) before and (**c**,**d**) after the sulfur supply, relative to the expression of ubiquitin. Bars show the mean of the biological replicates ± SE, asterisk (*) indicates the statistically significant difference between the sampling and the respective control at *p* < 0.05.

The expression levels of *ZmTOM1* in the roots of NM plants were significantly downregulated on day 45 and remained reduced until day 60. The sulfur supply induced a further downregulation on day 61, whilst the next day the expression was strongly upregulated (Figure 5a,c). In the roots of M plants, the respective expression levels of *ZmTOM1* were constantly downregulated, both before as well as after the sulfur supply (Figure 5b,d). 

### 2.6. Data Meta-Analysis

The data on all the iron homeostasis components examined in this study, as well as in our previous works [5,6] were combined and further analyzed, and the comparative analysis is depicted in Table 1. In this analysis, the relative expression ratios of *ZmNAS3*, *ZmNAS1*, *ZmDMAS1*, *ZmTOM1* and *ZmYS1* in the leaves and roots of M plants were calculated using the respective values of NM plants as control. Even on day 30, the ferric iron chelation components were differentially regulated in the leaves of M plants, although there was no difference observed in the M roots on that day, except for a strong downregulation of *ZmTOM1*. As a matter of fact, *ZmTOM1* was the sole iron homeostasis component permanently downregulated in the roots and upregulated in the leaves of M plants throughout the experiment. On the other hand, all of the examined genes were downregulated in the M roots after the addition of sulfate to the nutrient solution. 

## 3. Discussion

### 3.1. Roles of Ferric Iron Chelation Components in Maize Leaves

Following its entry in the root central cylinder, iron is transported via the xylem vessels to be utilized in the aerial parts. The dominant bound iron forms in the xylem sap of graminaceous plants were found to be of two types: ferric iron-citrate, along with various ferric iron-phytosiderophores; DMA and citrate were present in large concentrations in the xylem sap from rice and maize [4]. The iron deficiency-inducible genes of barley have been found to be expressed almost exclusively in roots, whereas many iron deficiency-inducible genes in rice were expressed in both roots and shoots [3]. In the shoot tissues, the *OsDMAS1* promoter-GUS analysis showed an expression in vascular bundles, specifically under iron-deficient conditions. No GUS expression was detected in the leaves of iron-sufficient plants, whereas under iron-deficient conditions, a GUS activity was detected in the phloem sieve tubes and companion cells, as well as in the xylem parenchyma cells of the large vascular bundles [3]. A similar study assessing the tissue specific localization of *OsTOM1* showed the same GUS reporter gene expression pattern in the vascular bundles of the leaves of iron-deficient plants [7]. In the case of YS1, the expression of *HvYS1* was only specifically induced by iron deficiency in barley roots, whereas *ZmYS1* was expressed in maize in the leaf blades and sheaths, crown, and seminal roots [8,9]. The fact that ZmYS1 was expressed in the leaves led to the hypothesis that it must have additional functions that are not related to iron uptake from the soil. The expression of ZmYS1 was only found in the leaves of iron deficient maize plants, which are thus producing DMA. In those iron-starved plants, the ferric iron-DMA substrate for ZmYS1 could arrive to the leaves following transport from the roots through the xylem.

In this study, M maize plants presented an early response in regulating iron homeostasis in the young expanding leaves (Table 1). The young expanding leaves were chosen as strong sinks of iron, whilst AM symbiosis was chosen as a treatment that forces the plant to optimize its photosynthetic efficiency, in order to feed the fungus. Ferric iron chelation components were differentially regulated in the leaves of M plants even on day 30, when the AM symbiosis was not yet visibly established. The roots of the maize plants were colonized by the fungus on day 45. In view of this, on day 30 the plant was already prepared for the upcoming symbiosis, and its efforts to maintain an efficient iron homeostasis in the young leaves was evident throughout the experimental period (Table 1). However, it seems that the genes related to iron uptake from the roots do have a role in the aerial plant parts. More specifically, it is hypothesized that the enzymes ZmNAS1, ZmDMAS1, ZmTOM1 and ZmYS1 have a role in the ferric iron chelation and/or unloading from the xylem vessels. 

**Figure 6:**
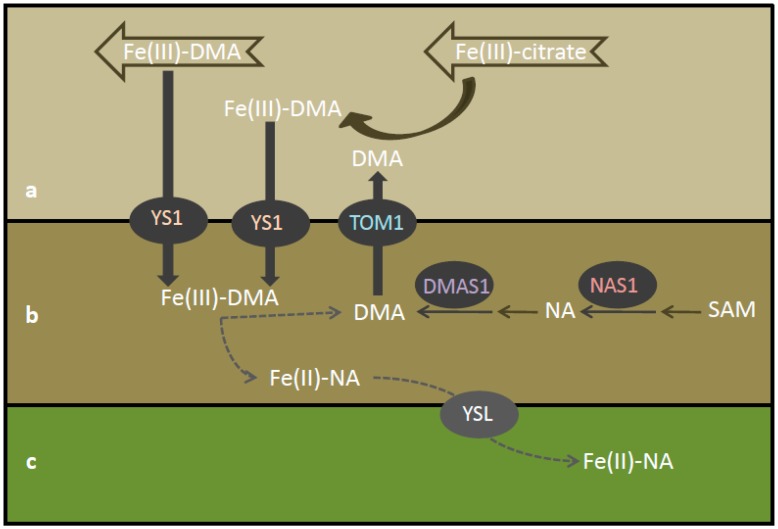
Conceptual model of iron xylem transport in young maize leaves, xylem-to-phloem iron exchange and iron re-translocation to younger tissues. The processes are considered to take place in the large vein of a maize leaf: (**a**) xylem vessel, (**b**) xylem parenchyma cell, and (**c**) phloem. DMA: deoxymugineic acid, DMAS: DMA synthase, NA: nicotianamine, NAS: nicotianamine synthase, SAM: S-adenosyl-methionine, TOM: DMA efflux transporter, YS: yellow stripe transporter, and YSL: yellow stripe like transporter.

It is hypothesized that DMA synthesized in the xylem parenchyma cells (through *ZmNAS1* and *ZmDMAS1*) is secreted to the xylem vessels (through *ZmTOM1*). The xylem sap in maize may contain ferric iron-citrate and ferric iron-DMA coming up from the roots. The secreted DMA chelates ferric iron delivered there in the form of ferric iron-citrate, because the ferric iron-DMA chelate has a higher stability than the ferric iron-citrate [10]. The resulting ferric iron-DMA, as well as the root-originated ferric iron-DMA in the xylem, may then enter the neighboring xylem parenchyma cells via the ZmYS1 transporter. The chelated iron may then be transported throughout the leaf and plant body via YSL transporters, for an efficient iron remobilization (Figure 6).

### 3.2. Arbuscular Mycorrhizal Fungus Provides Iron to M Plants 

The expression patterns of the examined genes were completely different among the various treatments, i.e., NM vs. M under sulfur deprivation and NM vs. M after sulfate supply. This fact holds true for the roots as well as for the leaves of the examined maize plants. This can be explained if we consider the hypothesis that NM plants suffer from iron deficiency throughout the treatment, whilst mycorrhizal colonization prevented iron deprivation responses due to a symbiotic iron uptake pathway [5].

The expression profile patterns of the examined genes in the roots of M and NM maize plants, both before as well as after sulfur supply, supported the previously suggested hypothesis that the fungus provides iron to the plants [5]. Despite the facts that: (a) before the addition of sulfur the concentration of total Fe in the shoots of NM plants decreased from day 45 to day 60, whilst the corresponding concentrations in M plants remained at the same levels and (b) five days after the addition of sulfur the concentration of total iron in M plants was higher than the corresponding concentration in NM plants [5], none of the genes related to iron uptake was overexpressed in the roots of M plants, neither during the sulfur deprivation period, nor after the supply of sulfur. Given this, in the M plants iron was probably delivered to the central cortex through the fungal arbuscules. Consequently, the M plants did not need to excrete DMA to their rhizosphere and *ZmTOM1* was strongly downregulated (Table 1, Figure 4 and Figure 5, [5]). 

Twenty four hours after the sulfur supply, a general transient downregulation of all the genes related to iron uptake was monitored in the roots of both M and NM plants (Figure 4 and Figure 5, [5,6]). Sulfate may act as a signal molecule regulating the expression of the iron uptake related genes in roots. It is suggested that the “massive entry” of inorganic sulfur into the root cells creates a transient “shortage of organic sulfur” condition, which is required for the DMA synthesis and that during this period of time the plants downregulate the whole DMA biosynthesis pathway, until organic sulfur will be again available.

Two days after the addition of sulfur to the nutrient solution, a diverse response was observed: the iron-uptake-related genes were upregulated in the roots of NM plants, whilst their expression in the M roots remained stable and downregulated from day 61 (Table 1, Figure 4 and Figure 5, [5]). 

Moreover, *ZmTOM1* was the only gene permanently overexpressed in the leaves and downregulated in the roots of M plants relative to NM ones, throughout the long-term experiment, even on day 30 when the M plants were not actually colonized by the fungus yet (Table 1). Therefore, the expression of *ZmTOM1* could be an early indicator of a mycorrhizal symbiotic relationship. 

## 4. Materials and Methods

### 4.1. Plant Material and Growth Conditions 

Mycorrhizal (M) and non-mycorrhizal (NM) maize plants were grown in pots with sterile river sand and practically insoluble FePO_4_ (500 mg per pot) in a long-term experiment, as previously described [5]. The plants were watered with a nutrient solution deprived of iron and sulfur and containing a minimum phosphorus concentration, in order to enhance the establishment of the symbiotic relationship with the AM fungus *Rhizophagus irregularis*. Iron was provided to plants throughout the experiment in the sparingly soluble form of FePO_4_. After a 60-day period of sulfur deprivation, sulfur was provided to the plants in the form of sulfate (2.5 mM CaSO_4_ 2H_2_O and 1 mM MgSO_4_ 7H_2_O). 

### 4.2. Plant Samplings 

The samplings were performed on days 30, 45, 60, 61 (24 h after the sulfur supply) and 62 (48 h after the sulfur supply) after sowing and 3 h after the onset of light. The sampling of day 60 took place before the addition of sulfur. The lateral roots, as well as two young expanding leaves, were immediately frozen in liquid nitrogen and stored at −80 °C until use. In each experiment, plant material from at least three biological replicates per treatment and sampling day was used [5]. 

### 4.3. Gene Expression Analysis

The gene expression analysis was conducted by means of Real-Time RT-PCR, as previously described [5]. The oligonucleotide primers used for RT-qPCR were as previously referred to [6]. The efficiency of each Real-Time RT-PCR reaction was calculated using the LinRegPCR software [11]. The following mathematical formula, from [12], was used for the calculations of the relative expression ratios of the target genes (*ZmDMAS1*, *ZmTOM1* and *ZmYS1*), whilst ubiquitin (*ZmUBQ*) was used as the reference gene:

(1) $$ratio=\frac{{\mathrm{Etarget}}^{\Delta CP\mathrm{target}\left(control-sample\right)}}{{\mathrm{Eref}}^{\Delta CP\mathrm{ref}\left(control-sample\right)}}$$

The samples of day 30 (for the samplings before the sulfur supply) or day 60 (for the samplings after the sulfur supply) of the respective treatment (NM or M) were used as control, unless otherwise specified. 

### 4.4. Data Meta-Analysis

Data from [5,6] were combined and further analyzed in order to make a comparative analysis, taking into consideration the data of all of the examined iron homeostasis components. The results of this analysis are depicted in Table 1.

### 4.5. Statistical Analysis

The experiment was performed two times under the same conditions and during two distinct time periods: autumn 2013 and spring 2014. The data were analyzed by t-test variance analysis with a two-tailed distribution and two-sample unequal variance to determine the significance of differences among the samplings.

## 5. Conclusions

It is suggested that the components related to iron uptake from the roots have a role in the ferric iron chelation and/or unloading from the xylem vessels in the aerial plant parts. Moreover, the expression of the gene codes for the DMA exporter *ZmTOM1* could be an early indicator for the establishment of a mycorrhizal symbiotic relationship. 

## Figures and Tables

**Table 1:** Relative expression ratios of *ZmNAS3*, *ZmNAS1*, *ZmDMAS1*, *ZmTOM1* and *ZmYS1* in the leaves and roots of mycorrhizal plants, before (days 30, 45, and 60) and after (days 61 and 62) the sulfur supply, relative to the expression of ubiquitin. Non-mycorrhizal plants were used accordingly as a control for the calculation of the relative expression ratios. The values show the mean of the biological replicates ± SE. Table cells highlighted with dark grey indicate an upregulation, and those in light grey indicate a downregulation of the respective gene, when the difference between the sampling and the respective control was statistically significant at *p* < 0.05.

		Days after Sowing				
	Gene	30	45	60	61	62
Leaves	*ZmNAS3*	0.91 ± 0.34	1.28 ± 0.12	**5.32 ± 1.25**	**2.85 ± 0.16**	**0.45 ± 0.11**
	*ZmNAS1*	**2.01 ± 0.27**	**2.69 ± 0.43**	1.13 ± 0.26	1.19 ± 0.20	**0.55 ± 0.03**
	*ZmDMAS1*	**0.61 ± 0.16**	1.37 ± 0.21	**1.80 ± 0.09**	**0.47 ± 0.03**	1.27 ± 0.15
	*ZmTOM1*	**1.99 ± 0.12**	**9.04 ± 1.68**	**4.12 ± 1.09**	**13.76 ± 3.54**	**3.31 ± 0.36**
	*ZmYS1*	**0.11 ± 0.06**	**1.76 ± 0.21**	**0.35 ± 0.14**	**0.28 ± 0.13**	**0.03 ± 0.01**
Roots	*ZmNAS3*	0.83 ± 0.13	**1.94 ± 0.11**	0.99 ± 0.07	1.33 ± 0.31	**0.67 ± 0.12**
	*ZmNAS1*	0.96 ± 0.22	**0.73 ± 0.08**	**0.63 ± 0.05**	**1.76 ± 0.12**	**0.41 ± 0.07**
	*ZmDMAS1*	1.12 ± 0.18	1.10 ± 0.25	0.85 ± 0.20	**0.48 ± 0.09**	**0.41 ± 0.15**
	*ZmTOM1*	**0.18 ± 0.10**	**0.38 ± 0.06**	**0.48 ± 0.14**	**0.45 ± 0.16**	**0.12 ± 0.02**
	*ZmYS1*	1.56 ± 0.31	**0.78 ± 0.04**	1.36 ± 0.19	1.05 ± 0.08	**0.12 ± 0.08**

## References

[1] Connorton J.M., Balk J., Rodriguez-Celma J. (2017). Iron homeostasis in plants—A brief overview. Metallomics.

[2] Li Y., Yu S., Strong J., Wang H. (2012). Are the biogeochemical cycles of carbon, nitrogen, sulfur, and phosphorus driven by the “Fe^III^-Fe^II^ redox wheel” in dynamic redox environments?. J. Soils Sediments.

[3] Bashir K., Inoue H., Nagasaka S., Takahashi M., Nakanishi H., Mori S., Nishizawa N.K. (2006). Cloning and characterization of deoxymugineic acid synthase genes from graminaceous plants. J. Biol. Chem..

[4] Ariga Τ., Hazama Κ., Yanagisawa S., Yoneyama T. (2014). Chemical forms of iron in xylem sap from graminaceous and non-graminaceous plants. Soil Sci. Plant Nutr..

[5] Chorianopoulou S.N., Saridis Y.I., Dimou M., Katinakis P., Bouranis D.L. (2015). Arbuscular mycorrhizal symbiosis alters the expression patterns of three key iron homeostasis genes, *ZmNAS1*, *ZmNAS3*, and *ZmYS1*, in S deprived maize plants. Front. Plant Sci..

[6] Saridis G.I., Chorianopoulou S.N., Katinakis P., Bouranis D.L., De Kok L.J., Hawkesford M.J., Haneklaus S.H., Schnug E. (2017). Evidence for regulation of the iron uptake pathway by sulfate supply in S-deprived maize plants. Sulfur Metabolism in Higher Plants—Fundamental, Environmental and Agricultural Aspects, Proceedings of the International Plant Sulfur Workshop, Goslar, Germany, 1–4 September 2015.

[7] Nozoye T., Nagasaka S., Kobayashi T., Takahashi M., Sato Y., Sato Y., Uozumi N., Nakanishi H., Nishizawa N.K. (2011). Phytosiderophore efflux transporters are crucial for iron acquisition in graminaceous plants. J. Biol. Chem..

[8] Roberts L.A., Pierson A.J., Panaviene Z., Walker E.L. (2004). Yellow Stripe1. Expanded roles for the maize iron-phytosiderophore transporter. Plant Physiol..

[9] Ueno D., Yamaji N., Ma J.F. (2009). Further characterization of ferric—Phytosiderophore transporters ZmYS1 and HvYS1 in maize and barley. J. Exp. Bot..

[10] Fodor F., Barton L.L., Abadía J. (2006). Heavy metals competing with iron under conditions involving phytoremediation. Iron Nutrition in Plants and Rhizospheric Microorganisms.

[11] Ruijter J.M., Ramakers C., Hoogaars W.M., Karlen Y., Bakker O., van den Hoff M.J., Moorman A.F. (2009). Amplification efficiency: Linking base line and bias in the analysis of quantitative PCR data. Nucleic Acids Res..

[12] Pfaffl M.W. (2001). A new mathematical model for relative quantification in real-time RT-PCR. Nucleic Acids Res..

